# Precise Correction of the *Pde6b*-L659P Mutation Causing Retinal Degeneration with Minimum Bystander Editing by Advanced Genome Editing Tools

**DOI:** 10.34133/research.0770

**Published:** 2025-07-02

**Authors:** Zhiquan Liu, Siyu Chen, Yang Sun

**Affiliations:** ^1^ Department of Ophthalmology, Stanford University School of Medicine, Palo Alto, CA 94304, USA.; ^2^ Palo Alto Veterans Administration, Palo Alto, CA, USA.

## Abstract

Recently developed base editing (BE), prime editing (PE), and click editing (CE) technologies enable precise and efficient genome editing with minimal risk of double-strand breaks and associated toxicity. However, their effectiveness in correcting real disease-causing mutations has not been systematically compared. Here, we aim to evaluate the potential of BE, PE, and CE technologies in rescuing the retinal degeneration-causing *Pde6b* (c.1976T>C, p.L659P) mutation. This site is prone to bystander effects, making it an ideal model for comparing the editing outcomes of these 3 novel technologies, particularly their editing precision. We optimized BE, PE, and CE systems in vitro using *Pde6b*-L659P cell models and compared their editing via deep sequencing. BE and PE had similar efficiency, but PE was the most precise, minimizing bystander edits. CE had lower efficiency and higher indel rates, needing further optimization. Using the optimal PE system for in vivo electroporation in *Pde6b*-L659P mice, we achieved 12.4% targeted repair with high precision, partially rescuing retinal degeneration. This study demonstrates proof of concept for the precise correction of the *Pde6b*-L659P mutation causing retinal degeneration using BE, PE, and CE tools. The findings offer valuable insights into the future optimization of precision gene editing techniques and their potential translational applications.

## Introduction

Most genetic variants associated with human diseases, including retinal disorders, are point mutations, also known as single-nucleotide polymorphisms (SNPs) [1,2]. For instance, in the context of retinal degeneration, point mutations in genes such as *Pde6b* can trigger the progressive degeneration of photoreceptor cells, ultimately resulting in vision loss [3,4]. Retinal degeneration is a group of inherited disorders characterized by progressive loss of photoreceptors and irreversible vision impairment [3]. Despite recent advances in gene therapy, most notably the Food and Drug Administration approval of voretigene neparvovec for *Rpe65*-associated Leber congenital amaurosis, most forms of retinal degeneration remain untreatable due to the genetic heterogeneity and challenges in achieving precise and durable correction [5,6]. These unmet clinical needs highlight the urgent demand for next-generation therapeutic strategies that are both versatile and precise.

The advent of innovative CRISPR genome editing technologies, exemplified by base editing (BE), prime editing (PE), and click editing (CE), has revolutionized the precise correction of disease-causing point mutations at the DNA level [7–9]. By enabling targeted repairs without inducing double-strand breaks, these advanced techniques offer a promising strategy for tackling the root causes of genetic disorders [7]. These 3 genome editing technologies share the ability to convert one DNA base pair into another, yet they operate through distinct molecular mechanisms. BE employs a catalytically impaired Cas9 nickase (nCas9) fused to a deaminase enzyme, enabling the targeted chemical modification of specific bases [1]. For instance, cytosine base editors (CBEs) convert cytosine (C) to thymine (T), while adenine base editors (ABEs) convert adenine (A) to guanine (G) [10–12]. PE, in contrast, utilizes a reverse transcriptase (RT) in conjunction with a prime editing guide RNA (pegRNA) to transcribe the desired genetic modifications from an RNA template into DNA, integrating them into the genome [13,14]. CE, the most recent of the three, harnesses DNA polymerase to incorporate editing information from a single-stranded DNA (ssDNA) template, mimicking the natural process of DNA replication [8,15–17]. However, despite their potential, these technologies—particularly the emerging CE approach—have yet to be rigorously tested or systematically compared in the context of real disease-causing mutations. Their efficiency, precision, and therapeutic viability still require further investigation and optimization.

**Fig. 1.**
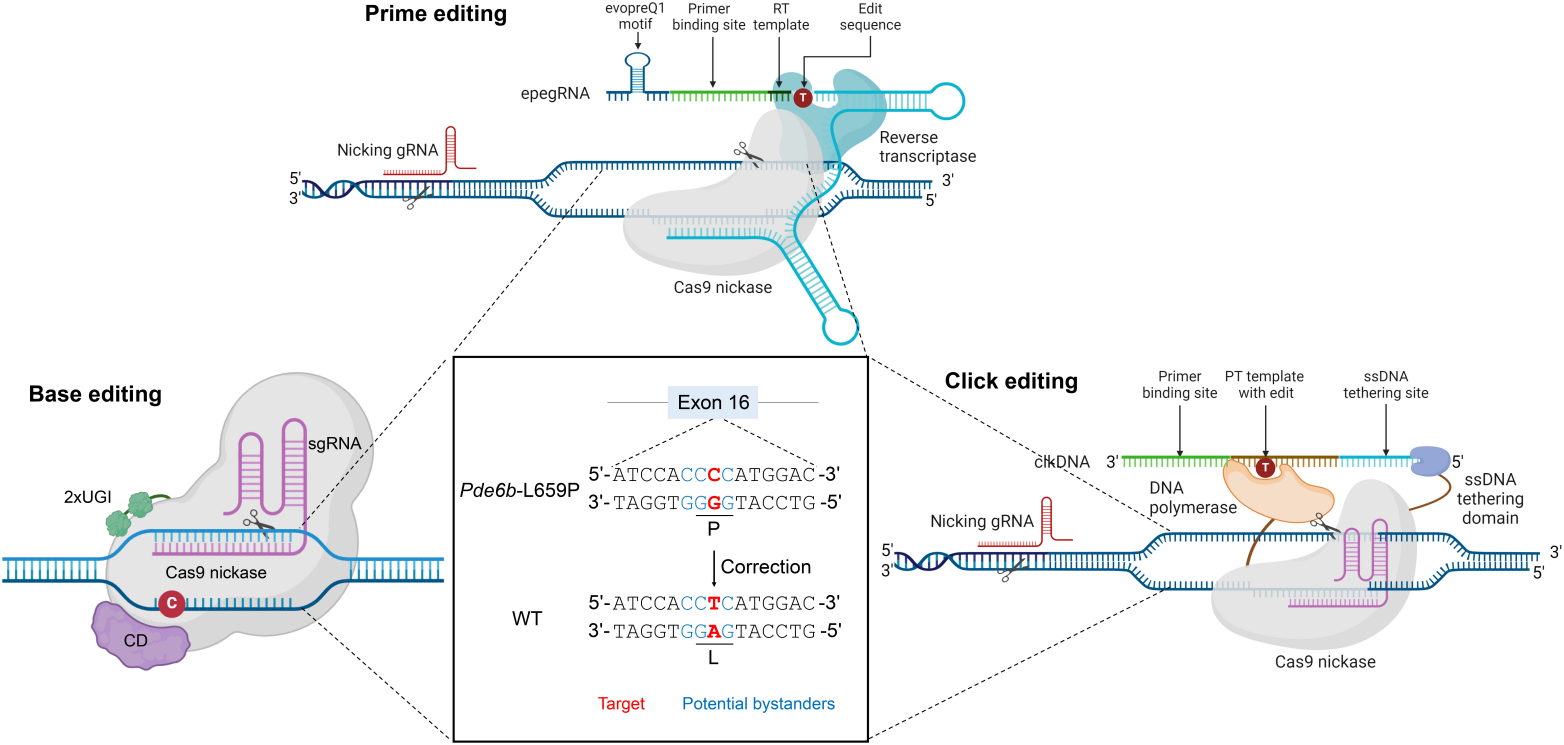
Schematic representation of the precise rescue of the *Pde6b*-L659P point mutation using BE, PE, and CE. Red indicates the target base, and blue indicates the bystander base. Due to the presence of 4 consecutive Cs at this locus, using gene editing tools for repair is prone to undesired bystander editing, making it suitable for comparing editing precision. The schematic diagram was created by biorender.com.

For the correction of point mutations, precision is crucial, as nontarget bystander edits on DNA may lead to unpredictable effects during mRNA transcription and protein translation [1]. Here, we selected a retinal degeneration-associated *Pde6b* (c.1976T>C, p.L659P) mutation to perform a parallel evaluation of the BE, PE, and CE systems [18] (Fig. 1). Notably, this mutation resides within a sequence of 4 consecutive cytosines, making it particularly susceptible to unintended bystander edits at neighboring C bases outside the target site (Fig. 1). This characteristic makes it an ideal site for assessing the precision of gene editing technologies. Using an in vitro cell model carrying the same point mutation, we systematically compared the editing efficiency, precision, and off-target effects of BE, PE, and CE at the *Pde6b* locus. Additionally, we validated the therapeutic potential of the most optimal PE system in an in vivo mouse model [18]. These findings provide valuable insights for the refinement and future application of BE, PE, and CE technologies in gene therapy.

## Results

### Establishment of a reliable cell model for in vitro screening

The *Pde6b*^nmf137^ mice, carrying a missense mutation c.1976T>C, p.L659P (hereafter referred to as the *Pde6b*-L659P mutation) in the *Pde6b* gene, exhibit severe photoreceptor degeneration and serve as a valuable model for studying and treating retinal degeneration [18] (Fig. 1). We aimed to perform a parallel comparison of the BE, PE, and CE systems at this disease-associated *Pde6b*-L659P mutation site. Before applying these systems in mouse in vivo studies, a reliable in vitro model is needed to optimize their parameters and achieve the most efficient editing system. To overcome this challenge, we utilized the ABE system (ABE8e-nSpRY) [19] to introduce the *Pde6b*-L659P mutation into the mouse Neuro-2a (N2a) cell line (Fig. S1A). After transfecting the ABE system, bulk sequencing results revealed that up to 45% of the desired target mutation was successfully introduced (Fig. S1B). However, this was accompanied by approximately 25% of 2 unintended bystander mutations (Fig. S1B). To address this, we screened edited single-cell colonies and ultimately obtained a stable N2a cell line carrying only the homozygous *Pde6b*-L659P mutation without bystander mutations (Fig. S1C). This mutation was completely identical to that in *Pde6b* mutant mice (Fig. S1C). Therefore, this endogenously mutated N2a cell model can be used for in vitro screening and comparison of different gene-editing systems for their repair effects.

### Optimization of the CBE system for *Pde6b*-L659P mutation correction

**Fig. 2.**
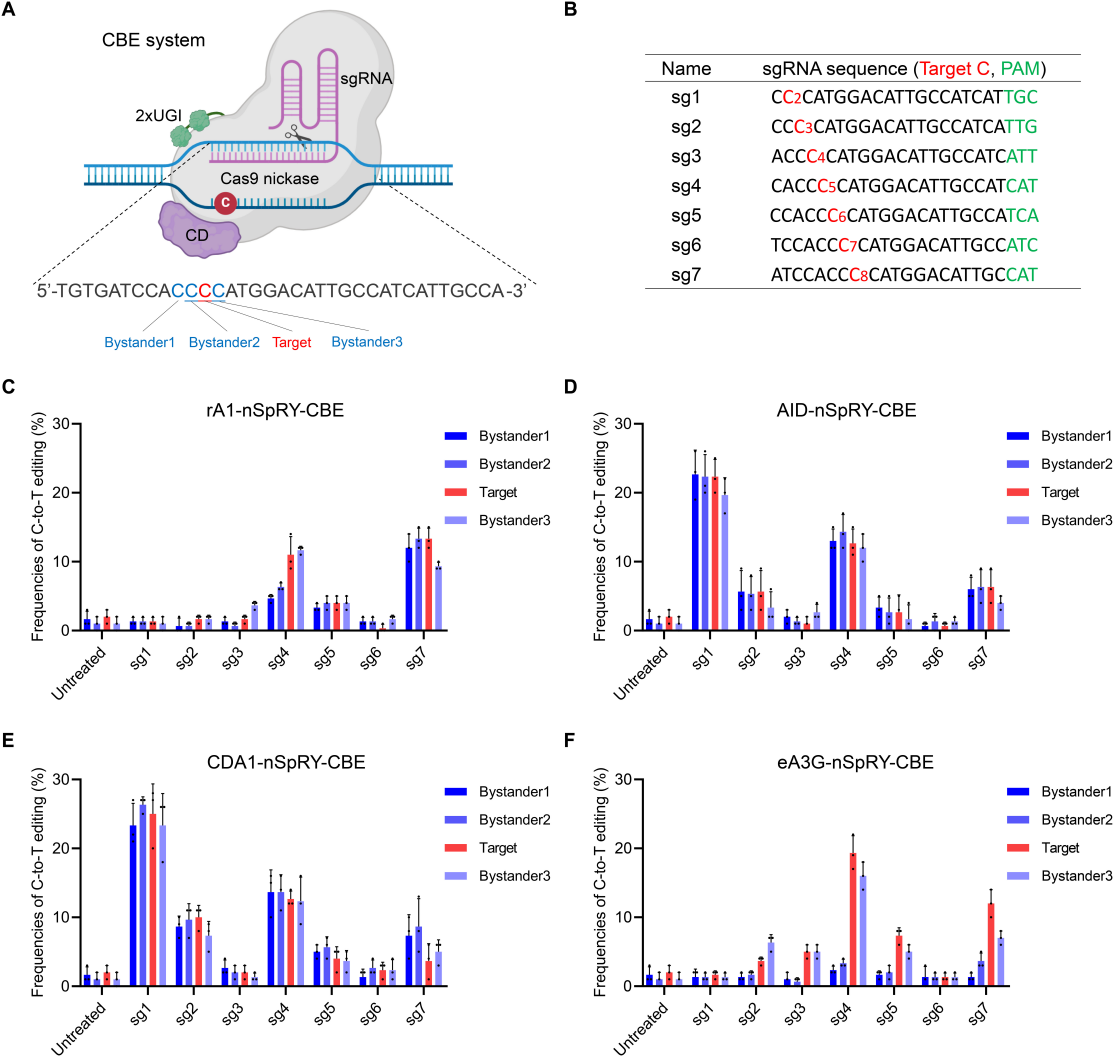
In vitro screening of CBE systems for *Pde6b*-L659P mutation correction. (A) Schematic representation of repairing *Pde6b*-L659P mutation by the CBE system (created by biorender.com). Bystander1 to 3 represent the C bases surrounding the target C that are easily edited simultaneously, leading to bystander mutations. (B) The 7 sgRNA sequences used for CBE system. Target base, red; PAM sequence, green. (C to F) Editing efficiencies of 7 tested sgRNAs with rA1-nSpRY-CBE (C), AID-nSpRY-CBE (D), CDA1-nSpRY-CBE (E), and eA3G-nSpRY-CBE (F) in the N2a cell model (*n* = 3 biologically independent experiments).

Since this *Pde6b*-L659P mutation is a T-to-C substitution, we hypothesized that the CBE system could be used for C-to-T conversion rescue. The CBE system consists of 2 components: the CBE vector and a single guide RNA (sgRNA) (Fig. 2A). Due to the absence of a classic NGG PAM sequence near the target site, we employed a near-PAMless nSpRY Cas9-mediated CBE, which theoretically recognizes all NNN PAMs (NRN > NYN, R = A/G, Y = C/T) [20]. For CBE vectors, we first tested the nSpRY-CBEs with 3 conventional cytosine deaminases (CDs, including rA1, AID, and CDA1) (Fig. S2A). For sgRNAs, we designed 7 sgRNAs with NNN PAMs, ensuring that the target C was positioned within the primary C2 to C8 editing window (Fig. 2B). To evaluate the BE performance, we introduced CBE vectors carrying sgRNAs 1 through 7 into the N2a cell model and measured the editing outcomes using Sanger sequencing. As a result, although all 3 CBEs achieved effective C-to-T editing at the target site, they also induced a high proportion of bystander edits (Fig. 2C to E). As expected, the presence of multiple adjacent Cs around the target site posed a challenge for precise editing. To address this issue, we tested eA3G-nSpRY-CBE, a system developed by us and other groups (Fig. S2A) [21–23]. This CBE utilizes an engineered A3G (eA3G) deaminase with preferential editing activity in CC motifs (where the preferentially deaminated C is underlined), which could potentially achieve better editing performance at this *Pde6b*-L659P site within CC contexts. Notably, eA3G-nSpRY-CBE in combination with sg4 achieved an optimal 19.3% targeted editing, with 16.0% bystander3 editing, and no noticeable bystander1 or bystander2 editing (Fig. 2E and Fig. S3A). Theoretically, due to the redundancy of the genetic code, bystander3 editing does not alter the intended amino acid correction (remaining p.P659L) and is therefore deemed acceptable (Fig. S3B). In addition to nSpRY-CBEs, we also tested the performance of these 4 deaminase-mediated nSpNG-CBEs, which are widely used systems capable of recognizing NGN PAMs (Fig. S2B) [24]. However, the results showed that the performance of nSpNG-CBEs did not outperform nSpRY-CBEs (Fig. S4A to D). Additionally, we tested a YE1-rA1-CBE with a narrowed editing window [25], but its efficiency was lower than that of rA1-CBE (Fig. S4E and F). Overall, after screening 10 CBE vectors and 7 sgRNAs in vitro, the combination of eA3G-nSpRY-CBE and sg4 achieved the most efficient targeted editing with the lowest unwanted bystander editing, making it the optimal CBE system for repairing the *Pde6b*-L659P mutation.

### Screening PE approaches for efficient *Pde6b*-L659P mutation correction

**Fig. 3.**
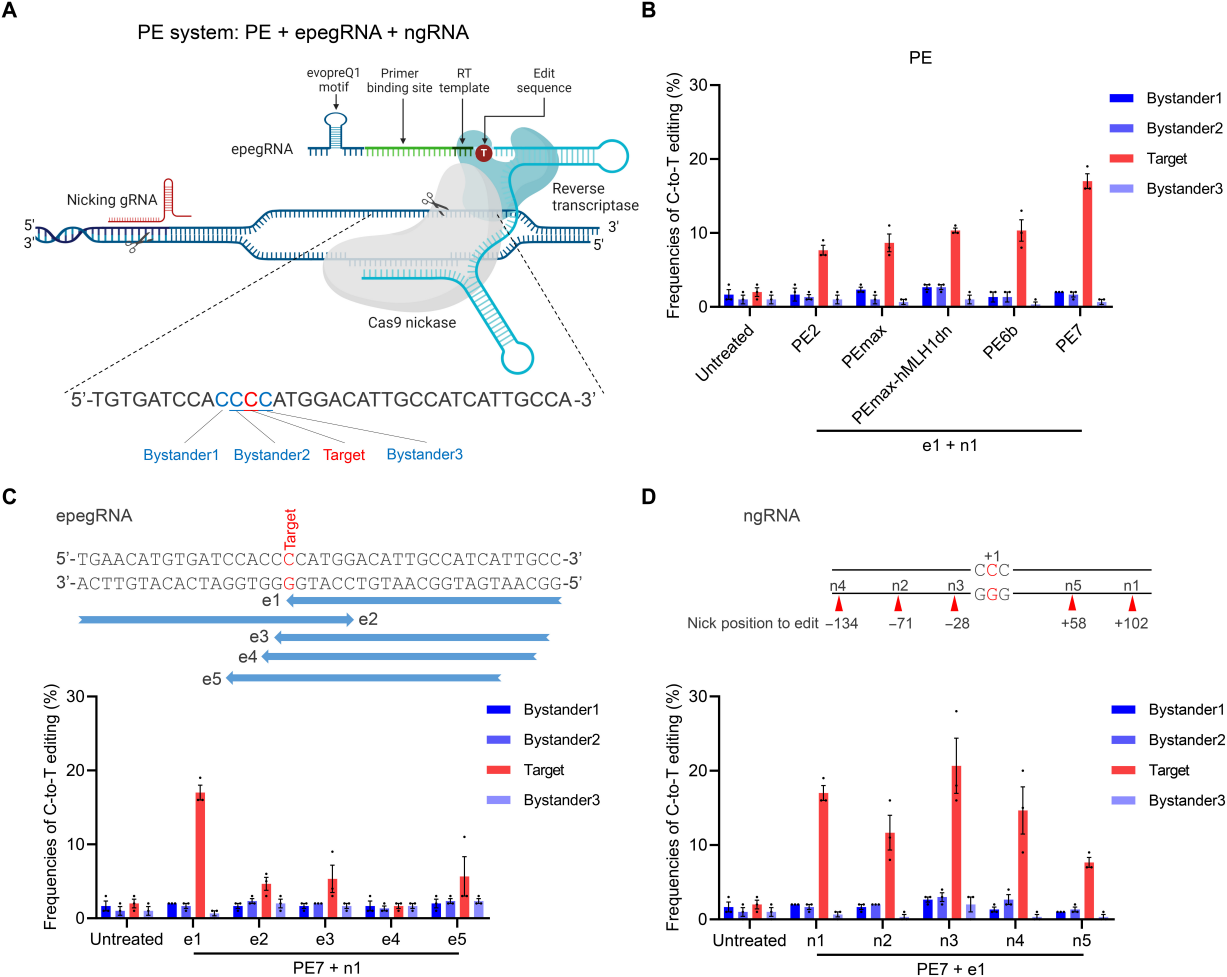
In vitro screening of PE systems for *Pde6b*-L659P mutation correction. (A) Schematic representation of repairing *Pde6b*-L659P mutation by the PE system (created by biorender.com). Bystander1 to 3 represent the C bases surrounding the target C that are easily edited simultaneously, leading to bystander mutations. (B) Comparison of editing efficiencies of 5 PE vectors in the N2a cell model. (C) Comparison of editing efficiencies of 5 different epegRNAs in the N2a cell model. (D) Comparison of editing efficiencies of 5 different ngRNAs in the N2a cell model. The ngRNAs have different nick positions (*n* = 3 biologically independent experiments).

Subsequently, we investigated the feasibility of using novel PE systems to correct the *Pde6b*-L659P mutation. Theoretically, PE offers greater precision than BE, as it directly installs the desired edit using a programmable template, thereby minimizing unintended bystander mutations [13,26,27]. The commonly used PE system typically consists of 3 components: the PE vector, engineered prime editing guide RNA (epegRNA) [14], and nicking guide RNA (ngRNA) (Fig. 3A). Following established PE experimental protocols [28], we systematically designed and tested 5 PE vectors (PE2 [13], PEmax [29], PEmax-hMLH1dn [29], PE6b [30], and PE7 [31]), along with 5 epegRNAs (e1 to e5) and 5 ngRNAs (n1 to n5) featuring diverse nonedited strand nicks (Fig. S5). These epegRNA and ngRNA designs were guided by recommendations from PE design web tools to optimize editing efficiency [32,33]. Evaluation in the N2a cell model revealed that the latest PE7 vector achieved 17% targeted editing with no noticeable bystander edits, outperforming all other PE vectors (Fig. 3B and Fig. S5). Among the 5 tested epegRNAs, e1 exhibited the highest editing efficiency, markedly surpassing the other 4 (Fig. 3C). Similarly, for the 5 ngRNAs, n1, n3, and n4 demonstrated strong efficiency, with n3 achieving the highest at 20.6% (Fig. 3D). In summary, in vitro screening results demonstrated that the combination of PE7 + e1 + n3 achieved the highest targeting efficiency with minimal bystander edits, showing greater precision than CBE for efficient *Pde6b*-L659P mutation correction.

### Evaluation of the CE system for *Pde6b*-L659P mutation repair

**Fig. 4.**
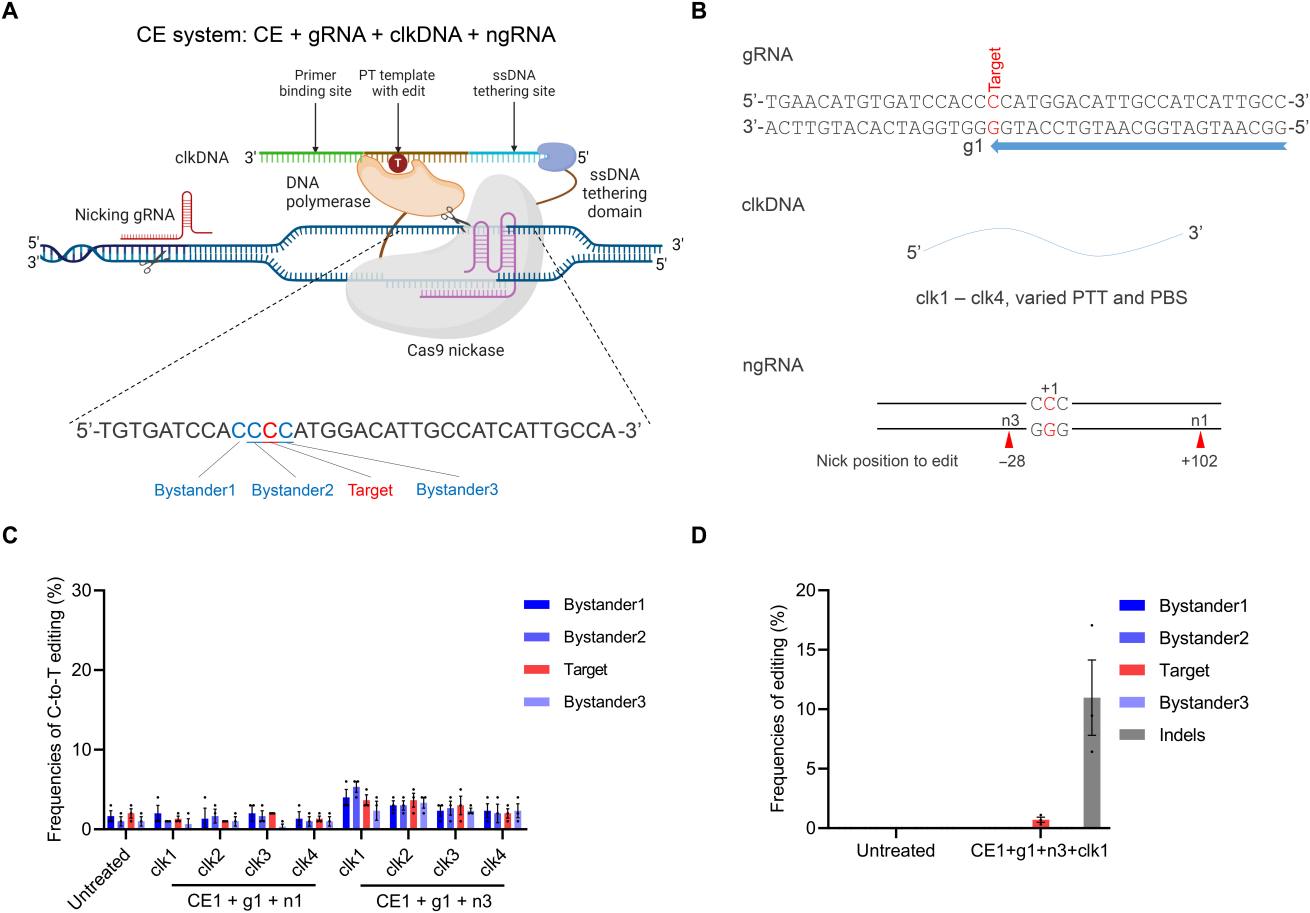
In vitro screening of CE systems for *Pde6b*-L659P mutation correction. (A) Schematic representation of repairing *Pde6b*-L659P mutation by the CE system (created by biorender.com). Bystander1 to 3 represent the C bases surrounding the target C that are easily edited simultaneously, leading to bystander mutations. (B) Schematic representation of CE-gRNA, clkDNAs, and ngRNAs designed for *Pde6b*-L659P mutation correction. A total of 4 clkDNAs with varied lengths of PTT or PBS and 2 ngRNAs with varied nicking locations were evaluated. (C) Comparison of editing efficiencies across different CE system combinations in the N2a cell model analyzed by Sanger sequencing. (D) Editing efficiencies of optimal CE system in the N2a cell model analyzed by deep sequencing. (*n* = 3 biologically independent experiments).

CE is a newly developed next-generation precise genome editing technology, following BE and PE [8]. In principle, CE combines DNA-dependent polymerase (DDP) with RNA-programmable nCas9 to enable precise genome editing by covalently linking user-defined edits to targeted genomic sites using click DNA (clkDNA) templates. A typical CE system consists of 4 components: CE vector, target CE-gRNA, clkDNA, and ngRNA (Fig. 4A). Here, CE-gRNA and clkDNA are roughly analogous to epegRNA in PE, while ngRNA mirrors the ngRNA in PE, functioning to nick the nontarget strand and enhance efficiency. Given the design similarities between CE and PE, we directly applied the most efficient design parameters from PE to develop the CE system. We designed and tested 1 gRNA (CE-g1), 2 ngRNAs (n1 and n3), and 4 clkDNAs (clk1 to clk4) in the N2a cell model (Fig. 4B). However, Sanger sequencing results showed that none of the tested CE systems achieved obvious targeted editing; there appeared to be a certain proportion of indels, indicating the need for more precise sequencing analysis (Fig. 4C). Therefore, we performed deep sequencing analysis on the optimal CE1 + g1 + n3 + clk1 combination. The results showed that the CE system achieved a low efficiency of 0.7% on-target editing, but was accompanied by a higher efficiency of 10.9% indels (Fig. 4D). Previous studies have also indicated that current CE systems have low editing efficiency at certain sites and induce high levels of indels [8]. Our results demonstrated that the CE system can achieve targeted repair at the *Pde6b*-L659P locus, but further optimization is required to enhance editing efficiency and reduce indels.

### Comparative analysis of CBE, PE, and CE systems for *Pde6b*-L659P mutation repair

**Fig. 5.**
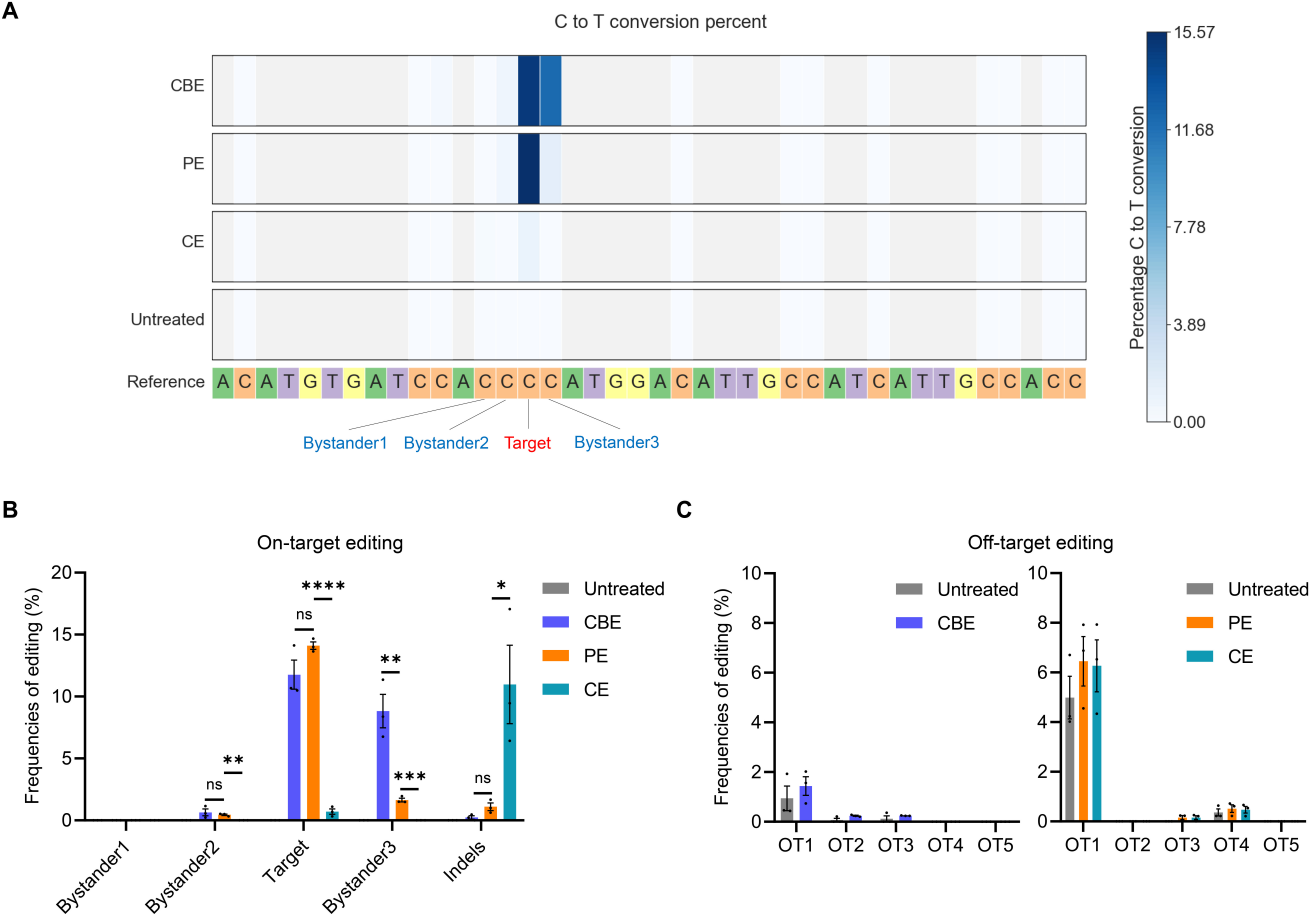
Parallel comparison of optimal CBE, PE, and CE systems for *Pde6b*-L659P mutation correction by deep sequencing. (A) Heat maps showing C-to-T conversion efficiency of CBE, PE, and CE systems for *Pde6b*-L659P mutation repair in the N2a cell model. Bystander1 to 3 represent the C bases surrounding the target C that are easily edited simultaneously, leading to bystander mutations. (B) Comparison of target editing, bystander editing, and indel frequencies among CBE, PE, and CE systems at the *Pde6b* on-target site. *P < 0.05, **P < 0.01, ***P < 0.001, ****P < 0.0001, ns: nonsignificant difference. (C) Evaluation of off-target editing frequencies of CBE, PE, and CE systems at the top 5 predicted off-target sites. OT, off-target (*n* = 3 biologically independent experiments).

Through a series of in vitro screenings in the N2a cell model, we have preliminarily identified the CBE, PE, and CE systems that exhibited the best performance in repairing the *Pde6b*-L659P mutation. However, their detailed editing characteristics still require further in-depth evaluation and comparison. Therefore, we performed deep sequencing on the genomic DNA of cells edited by the optimal CBE, PE, and CE systems to conduct a detailed and parallel comparison of their editing efficiency and precision. The deep sequencing results showed that CBE primarily edited the target and bystander3, while PE was more precise, mainly editing only the target, consistent with the previous Sanger sequencing results (Fig. 5A and Fig. S6). From a more detailed parallel comparison, PE and CBE exhibited similar repair efficiency on the target (14.1% versus 11.7%), but PE significantly reduced unwanted mutations at bystander3 (1.6% versus 8.8%) (Fig. 5B). In contrast, CE demonstrated lower editing efficiency on the target (0.7%) and induced a higher rate of indels (10.9%) (Fig. 5A and B and Fig. S6). Additionally, we performed deep sequencing on the top 5 predicted off-target sites for these 3 systems [34]. No obvious off-target effects were detected in any of the systems beyond the baseline seen in the controls, confirming the high fidelity of these genome editing systems, which aligns with previous studies [8,21,35,36] (Fig. 5C). Although, as mentioned earlier, the mutation at bystander3 is theoretically unlikely to affect the correct repair of the amino acid, its potential impact on the DNA and mRNA context remains difficult to assess. Therefore, given the similar on-target editing efficiency, we selected the more precise PE system for the next step of in vivo repair evaluation.

### In vivo correction of *Pde6b*-L659P mutation by PE plasmid electroporation

**Fig. 6.**
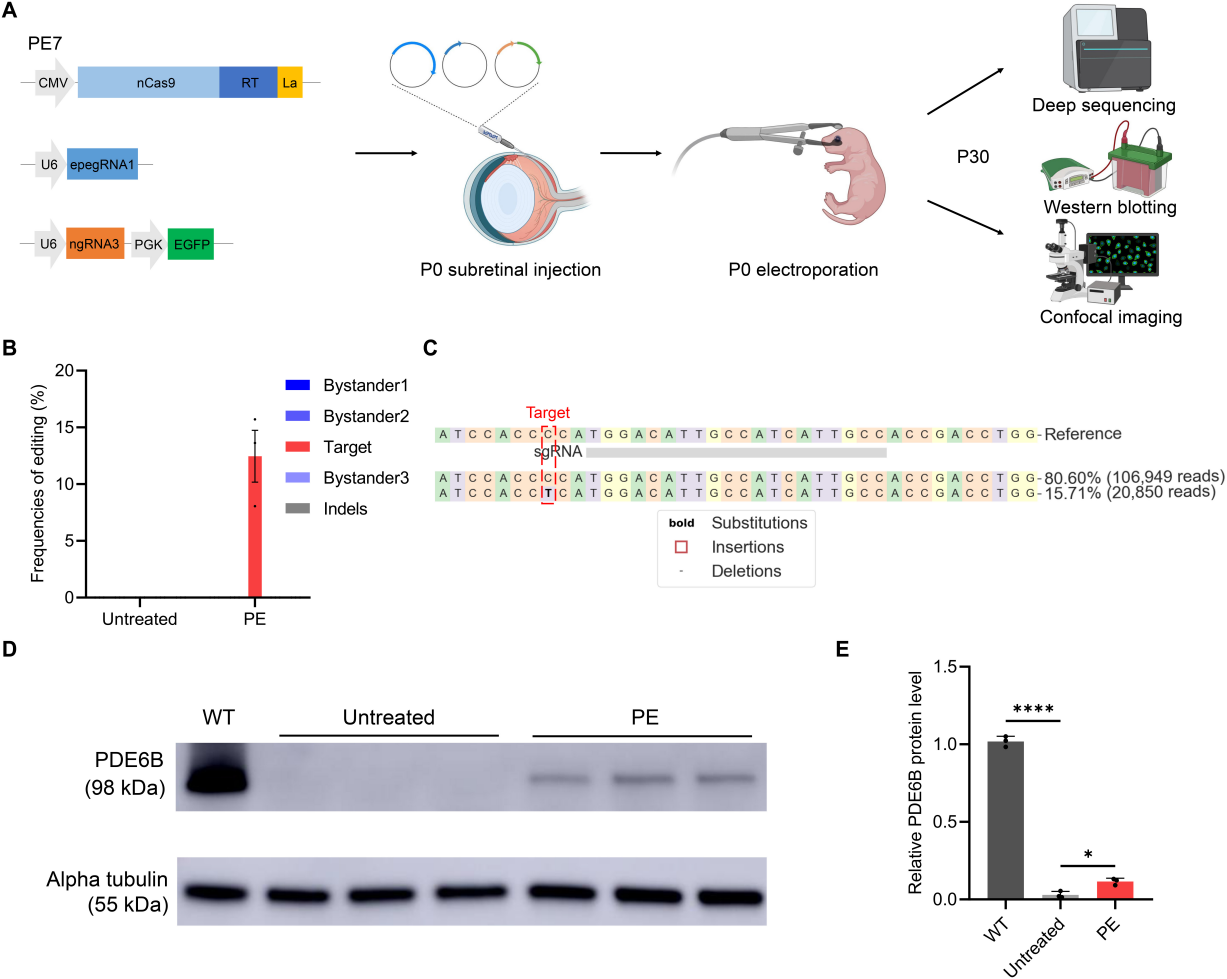
In vivo correction of the *Pde6b*-L659P mutation by electroporation of the PE system. (A) Workflow of subretinal PE plasmid injection and electroporation at P0 (postnatal day 0), followed by rescue effect assessment at P30 (created with BioRender.com). (B) Editing efficiency of untreated and PE-electroporated mouse retinas at the *Pde6b*-L659P site (*n* = 3 eyes). (C) Representative deep sequencing result of PE-edited mouse retina at the *Pde6b*-L659P site. Target base, red dotted box. (D) Western blotting of PDE6B protein expression in WT mice, untreated and PE-treated *Pde6b*-L659P mice at P30. WT, wild type (*n* = 3 eyes). (E) Quantification of the relative PDE6B protein level normalized to alpha tubulin. *P < 0.05, ****P < 0.0001.

Building on the efficient and precise PE correction observed in vitro, we advanced to in vivo evaluation by delivering PE plasmids via electroporation. This well-established technique enables efficient DNA plasmid delivery into the neonatal mouse retina, facilitating the study of retinal gene function and the assessment of gene therapy efficacy [27,37,38]. To achieve this, we combined the 3 plasmids encoding the top-performing PE systems, PE7 + e1 + n3, and introduced them into the retinas of newborn *Pde6b*-L659P mice (Fig. 6A). The co-expression of enhanced green fluorescent protein (EGFP) served as a marker of successful electroporation. The electroporated mouse retinas were then dissected at P30, a stage when the retinas of *Pde6b*-L659P mice are fully degenerated [18]. Afterward, we used deep sequencing, Western blotting (WB), and confocal imaging techniques to evaluate the rescue effect (Fig. 6A). Remarkably, electroporation of PE plasmids achieved an average target editing efficiency of 12.4%, with no detectable bystander editing or indels, demonstrating the exceptional precision of the PE system in rescuing the *Pde6b*-L659P mutation in vivo (Fig. 6B and C). Additionally, the WB results showed that the retinas of *Pde6b*-L659P mice in the PE-treated group exhibited an obvious restoration of PDE6B protein expression, but it was not as high as in the wild-type (WT) mice group (~11% of WT levels) (Fig. 6D and E). In contrast, the untreated control group showed almost no PDE6B expression (Fig. 6D and E). The WB results strongly supported that PE-mediated repair of the *Pde6b*-L659P mutation at the DNA level successfully rescued PDE6B protein expression, demonstrating the efficacy of precise gene editing.

### Preservation of retinal photoreceptors in *Pde6b*-L659P mice by PE rescue

**Fig. 7.**
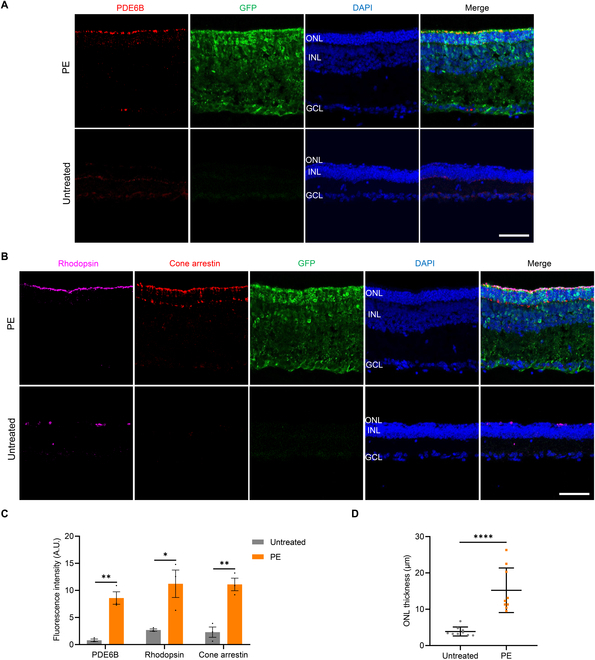
Preservation of retinal photoreceptors in *Pde6b*-L659P mice after PE treatment. (A) Representative immunofluorescence images of retinal sections stained with anti-PDE6B antibodies in untreated and PE-treated *Pde6b*-L659P mice at P30. Nuclei are counterstained with DAPI (blue), and GFP signal (green) marks successful electroporation. ONL, outer nuclear layer; INL, inner nuclear layer; GCL, ganglion cell layer. Scale bar: 50 μm. (B) Immunofluorescence analysis of rhodopsin and cone arrestin in untreated and PE-treated *Pde6b*-L659P mice at P30. Scale bar: 50 μm. (C) Quantification of the fluorescence intensities of PDE6B, rhodopsin, and cone arrestin in untreated and PE-treated *Pde6b*-L659P mice. *P < 0.05, **P < 0.01. (D) Quantification of ONL thickness in untreated and *Pde6b*-L659P mice (*n* = 3 eyes, 3 values per eye). ****P < 0.0001.

PDE6B protein is primarily expressed in the photoreceptor cells of the retina, specifically within the outer segment of rods and cones. It plays a crucial role in the phototransduction pathway, where it is involved in the conversion of light signals into electrical signals in the retina [3,4]. In *Pde6b*-L659P mutant mice, the photoreceptors gradually degenerate and, within approximately 1 month, are completely lost [18]. We performed immunofluorescence analysis on retinal sections to evaluate whether PE-mediated correction of the *Pde6b*-L659P mutation maintained photoreceptor integrity. Cryosections of retinas from P30 mice were immunolabeled with an anti-PDE6B antibody. The results demonstrated that in the PE-treated group, PDE6B protein expression was significantly restored and correctly localized to the outer segment of the retina (Fig. 7A and Fig. S7). Conversely, the untreated group exhibited almost no detectable PDE6B expression (Fig. 7A and Fig. S7). Next, we employed antibodies against rhodopsin, a rod-specific marker, and cone arrestin, a cone-specific marker, to assess the therapeutic impact of PE rescue on both rods and cones, the 2 main types of photoreceptors. In untreated retinas, the signals for rhodopsin and cone arrestin were low, as compared to the PE-treated retinas, both photoreceptor markers showed strong expression and were correctly localized—highlighting the remarkable preservation of both rods and cones (Fig. 7B). Furthermore, fluorescence intensity analysis confirmed that retinal photoreceptor cells were effectively preserved in the PE-treated retinas, in contrast to the pronounced degeneration observed in the control group (Fig. 7C). Additionally, at P30, untreated retinas showed only 1 to 2 layers of nuclei in the outer nuclear layer (ONL), while PE-treated retinas exhibited a considerably thicker ONL (Fig. 7A and B). Quantitative analysis of retinal sections showed that the ONL thickness in the PE group was 15.2 μm, a 4.0-fold increase compared to the untreated group (Fig. 7D). These findings collectively suggested that PE treatment effectively preserves retinal photoreceptors in *Pde6b*-L659P mice.

In addition, we used electroretinography (ERG) to measure the electrical activity of photoreceptors and optokinetic tracking response (OKR) to quantify visual acuity at P30. As a result, the PE-treated group showed a slight increase in scotopic ERG response compared to the untreated group (34.8 ± 5.8 μV vs. 16.0 ± 1.7 μV, at 1 cd⋅s/m^2^) (Fig. S8A and B). However, the OKR test indicated that PE plasmid electroporation did not lead to a notable recovery of visual function (Fig. S8C and D). We speculate that electroporation itself may cause structural damage to the eye, thereby affecting the restoration of visual function [37,39]. Therefore, using less invasive delivery methods such as adeno-associated virus (AAV) to administer PE may be a better therapeutic option in the future.

## Discussion

In this study, we used an N2a cell model that precisely simulates the endogenous *Pde6b*-L659P mutation to screen and optimize 3 representative precision genome editing tools: CBE, PE, and CE systems in vitro. We then performed a parallel comparison of the editing efficiency, precision, and off-target effects of the most optimal CBE, PE, and CE systems, finding that the PE system performed the best. Finally, we conducted a detailed evaluation of the therapeutic effect of PE in *Pde6b*-L659P mutant mice and found that PE exhibited effective on-target editing with high precision, successfully restoring PDE6B protein expression and partially rescuing the degeneration of photoreceptors. These results highlight the effectiveness of PE as a precise therapeutic tool and its utility as a high-precision genome editing technology.

We also evaluated the *Pde6b*-L659P mice at P60 following PE treatment to assess the long-term efficacy of the therapy. Efficient targeted editing was still detectable at this time point, and ERG recordings revealed a slight improvement in retinal responses (Fig. S9). Although both our findings and prior studies support the high specificity of PE-mediated editing [27,35,36,40], potential concerns such as toxicity from sustained overexpression of PE components, immune responses, and long-term safety risks warrant further investigation. Moreover, considering that the current in vivo editing efficiency of PE remains relatively low at approximately 12%, which may be insufficient to achieve a robust therapeutic effect, further optimization of the PE system is warranted. Future improvements could involve engineering the Cas9 and reverse transcriptase components, optimizing pegRNA design, or incorporating additional factors or peptides to enhance editing efficiency [41–43].

BE, PE, and CE all operate through distinct mechanisms to achieve precision genome editing. PE is more precise than BE because it allows for greater control over the editing process, minimizing bystander edits—undesired mutations at nearby sites—which are more common in BE [44]. Unlike BE, which uses a deaminase enzyme to convert bases within a predefined editing window and can lead to unintended bystander mutations, PE utilizes a reverse transcriptase to precisely substitute DNA sequences according to a programmable template, directly specifying the desired edit without relying on endogenous repair pathways. Based on our data, PE shows comparable efficiency to BE but with markedly better precision, making it more suitable for treating disease loci like the *Pde6b*-L659P mutation, which is prone to bystander editing. On the other hand, CE, while promising as a next-generation technique, currently has lower efficiency and higher rates of indels, requiring further refinement to enhance its therapeutic potential. The low efficiency and high indel rates observed with CE may result from suboptimal coordination between its components, such as DNA polymerase recruitment, CE-gRNA and clkDNA design, and endogenous DNA repair mechanisms. These mechanistic limitations highlight the need for further optimization. Notably, prime editing also faced similar challenges at its inception but has since been markedly improved through systematic engineering. With continued efforts to refine CE’s components and delivery strategies, it holds strong potential to evolve into a precise and efficient genome editing tool.

Here, we specifically optimized and compared BE, PE, and CE systems for the *Pde6b*-L659P mutation, a site prone to bystander edits, providing valuable insights into the application and refinement of precise genome editing tools. However, the sheer number of pathogenic point mutations makes it impractical to conduct such detailed screening for every mutation. In the future, leveraging artificial intelligence to analyze large-scale genomic editing data and predict the most efficient and precise tool for a given mutation could accelerate the advancement and generalizability of gene-editing therapies, streamlining the selection process and enhancing therapeutic outcomes.

Despite its high efficiency and precision, PE is limited by its large size, making it challenging to deliver using conventional AAV vectors. Splitting PE into multiple AAVs for co-delivery may reduce overall editing efficiency, posing a hurdle for therapeutic applications [27,45–48]. One potential solution is to engineer and evolve smaller versions of Cas9 and reverse transcriptase while maintaining editing efficiency, thereby reducing the overall size of the PE system [30,49,50]. Another approach is to explore alternative delivery platforms such as lipid nanoparticles and virus-like particles, which can accommodate larger payloads [51–56]. However, these delivery methods still require further optimization to improve their efficiency and specificity for precise genome editing applications.

This study systematically optimized and compared BE, PE, and CE systems for correcting the *Pde6b*-L659P mutation, a site susceptible to bystander editing, identifying PE as the most precise and efficient approach. Using the optimized PE system, we achieved 12.4% targeted editing with high fidelity in *Pde6b*-L659P mutant mice, successfully restoring PDE6B expression and partially rescuing photoreceptor degeneration. These findings highlight PE’s therapeutic potential and offer valuable insights for advancing precision genome editing strategies for genetic disorders.

## Materials and Methods

### Plasmid construction

The CBE plasmids used in this study, including rA1-nSpRY-CBE (#139999), rA1-nSpNG-CBE (#125617), YE1-nSpNG-CBE (#138159), CDA1-nSpNG-CBE (#125612), AID-nSp-CBE (#174696), A3G-nSp-CBE (#163636), and ABE8e-nSpRY (#185671), were obtained from Addgene. Additional CBE plasmids were generated through site-directed mutagenesis and DNA recombination cloning, using pre-existing plasmids as templates. These modifications were performed with the Fast Site-Directed Mutagenesis Kit (Vazyme, #C214) and the ClonExpress Ultra One Step Cloning Kit (Vazyme, #C115). For PE plasmids, the PE2 (#132775), PEmax (#180020), PEmax-hMLH1dn (#174828), PE6b (#207852), and PE7 (#214812) were obtained from Addgene. The epegRNA plasmids were constructed based on the pU6-tevopreq1-GG-acceptor (#174038), following published protocols [13,28]. For CE plasmids, the Click editor-CE1 (#208942) was obtained from Addgene. The clkDNAs were designed according to the original guidelines [8] and synthesized by Integrated DNA Technologies. The sequences of all gRNAs and clkDNAs are listed in Table S1.

### Cell culture and transfection

The N2a cell line was maintained in Dulbecco’s Modified Eagle’s Medium (Corning, #10013CV) supplemented with 10% fetal bovine serum (FBS) under standard conditions of 37°C with 5% CO₂. For transfection, cells were plated in 24-well plates and treated with PolyJet In Vitro DNA Transfection Reagent (SignaGen Laboratories, #SL100688). Specifically, 1.5 μl of PolyJet reagent was mixed with a total of 500 ng of BE, PE, or CE plasmids per well. After 72 h, cells were lysed for subsequent sequencing. Target sequences were amplified using primers listed in Table S2, and editing efficiency was assessed through Sanger sequencing, analyzed via EditR [57].

### N2a cell model generation

N2a cells were engineered to carry the *Pde6b* (c.1976T>C, p.L659P) mutation by transfecting them with ABE8e-nSpRY and ABE-gRNA-PGK-Puro plasmids. After transfection, cells underwent puromycin selection (2 μg/mL) for 5 days. Surviving cells were then diluted and plated into 96-well plates for further expansion. Individual colonies were isolated and verified by Sanger sequencing. A successfully mutated single-cell clone was expanded and used as the N2a cell model for subsequent in vitro screening.

### Animals

WT C57BL/6 mice (Charles River Laboratories, #027) and *Pde6b*^nmf137^ mutant mice (The Jackson Laboratory, #004766) were used in this study. This *Pde6b*^nmf137^ mutant mice were generated by ethylnitrosourea mutagenesis in C57BL/6J genetic background by The Jackson Laboratory [18]. The *Pde6b*^nmf137^ mutants showed extensive degeneration of the ONL of the retina starting at P16, and no ONL was left in the retina by P30 [18]. The *Pde6b*^nmf137^ mice used in this study were confirmed by Sanger sequencing to carry a homozygous *Pde6b*-L659P mutation, and retinal dissection further validated that they exhibited a clear retinal degeneration phenotype. Experimental procedures were conducted in accordance with protocol #32223, approved by the Institutional Animal Care and Use Committee of Stanford University School of Medicine.

### In vivo electroporation

The in vivo retinal electroporation in *Pde6b*-L659P neonatal mice (P0) was performed following established protocols [27,37,58]. In brief, pups were anesthetized by ice chilling, and their eyelids were gently opened using a 30-gauge needle. A 0.3- to 0.5-μl PE DNA solution (containing 1000 ng/μl of each plasmid and 0.1% Fast Green dye for visualization) was carefully delivered into the subretinal space using a FemtoJet 4i microinjector (Eppendorf, #5252000021). Following injection, the pup’s head was positioned between a 10-mm tweezer electrode, and five 80-V pulses (50 ms each, 950-ms intervals) were administered using a NEPA21 electroporator (Bulldog Bio). Subsequent EGFP expression was used as a marker of successful electroporation.

### Targeted deep sequencing

The detailed procedure has been previously published [27]. Briefly, genomic DNA was isolated from N2a cells or GFP-positive regions of electroporated mouse retinas. Potential off-target sites for CBE-sgRNA, PE-epegRNA, and CE-gRNA were identified using Cas-OFFinder [34]. Targeted deep sequencing was performed using the Amplicon-EZ service from Azenta Life Sciences, with data analysis conducted via CRISPResso2 [59]. The primers for on-target and off-target amplification are listed in Tables S2 and S3.

### Western blotting

The detailed procedure has been previously published [27]. Primary antibodies included anti-PDE6B (Proteintech, #22063-1-AP, 1:1000) and anti-alpha tubulin (Proteintech, #11224-1-AP, 1:5000) as a loading control. Chemiluminescence signals were captured directly using a digital imaging system (Amersha Imager 600). The images were quantitatively analyzed using Fiji ImageJ.

### Immunofluorescence analysis

The detailed procedure has been previously published [27]. The following primary antibodies were used: rabbit anti-PDE6B (Proteintech, #22063-1-AP, 1:500), mouse anti-rhodopsin (Abcam, #ab5417, 1:1000), and rabbit anti-cone arrestin (Millipore, #AB15282, 1:500). For secondary antibodies, Alexa-Fluor-555-conjugated anti-rabbit IgG (Invitrogen, #A21428, 1:500) and Alexa-Fluor-647-conjugated anti-mouse IgG (Invitrogen, #A-31571, 1:500) were used. Images were captured using a Zeiss LSM880 inverted confocal microscope. All images were acquired under nonsaturating conditions using identical acquisition settings to ensure comparability. Fluorescence intensity within defined regions of interest was quantitatively analyzed using Fiji (ImageJ). Mean fluorescence values were obtained through the integrated “Analyze > Measure” function.

### Electroretinography

The detailed procedure has been previously published [27]. Briefly, the ERG was performed with an ERG stimulator (Celeris, Diagnosys LLC) according to the manufacturer’s instructions. Mice were stimulated with flashes of 0.01, 0.1 and 1 cd⋅s/m^2^ light intensity.

### Optokinetic tracking response

The detailed procedure has been previously published [60,61]. Briefly, the OKR was assessed using the OptoMotry system (CerebralMechanics Inc.), a virtual-reality platform designed to swiftly quantify visuomotor behavior according to the manufacturer’s instructions.

### Statistical analysis

All data are expressed as mean ± SEM (standard error of the mean) of at least 3 individual determinations for all experiments. Statistical differences between the values were tested using 2-tailed unpaired Student’s *t* tests by GraphPad Prism software 8.0.1. The exact sample size was clarified in each figure legend.

## Data Availability

Deep sequencing data have been deposited in the National Center for Biotechnology Information (NCBI) Sequence Read Archive (SRA) database with BioProject accession code PRJNA1221314.
