# Supplementary material for: Precise Correction of the Pde6b-L659P Mutation Causing Retinal Degeneration with Minimum Bystander Editing by Advanced Genome Editing Tools
Source: Research (Wash D C). 2025 Jul 2;8:0770. doi: 10.34133/research.0770 (PMC12220932; doi:10.34133/research.0770)

Supplementary information

Precise correction of the *Pde6b*-L659P mutation causing retinal degeneration with minimum bystander editing by advanced genome editing tools

Zhiquan Liu[1], Siyu Chen[1], Yang Sun[1,2]*

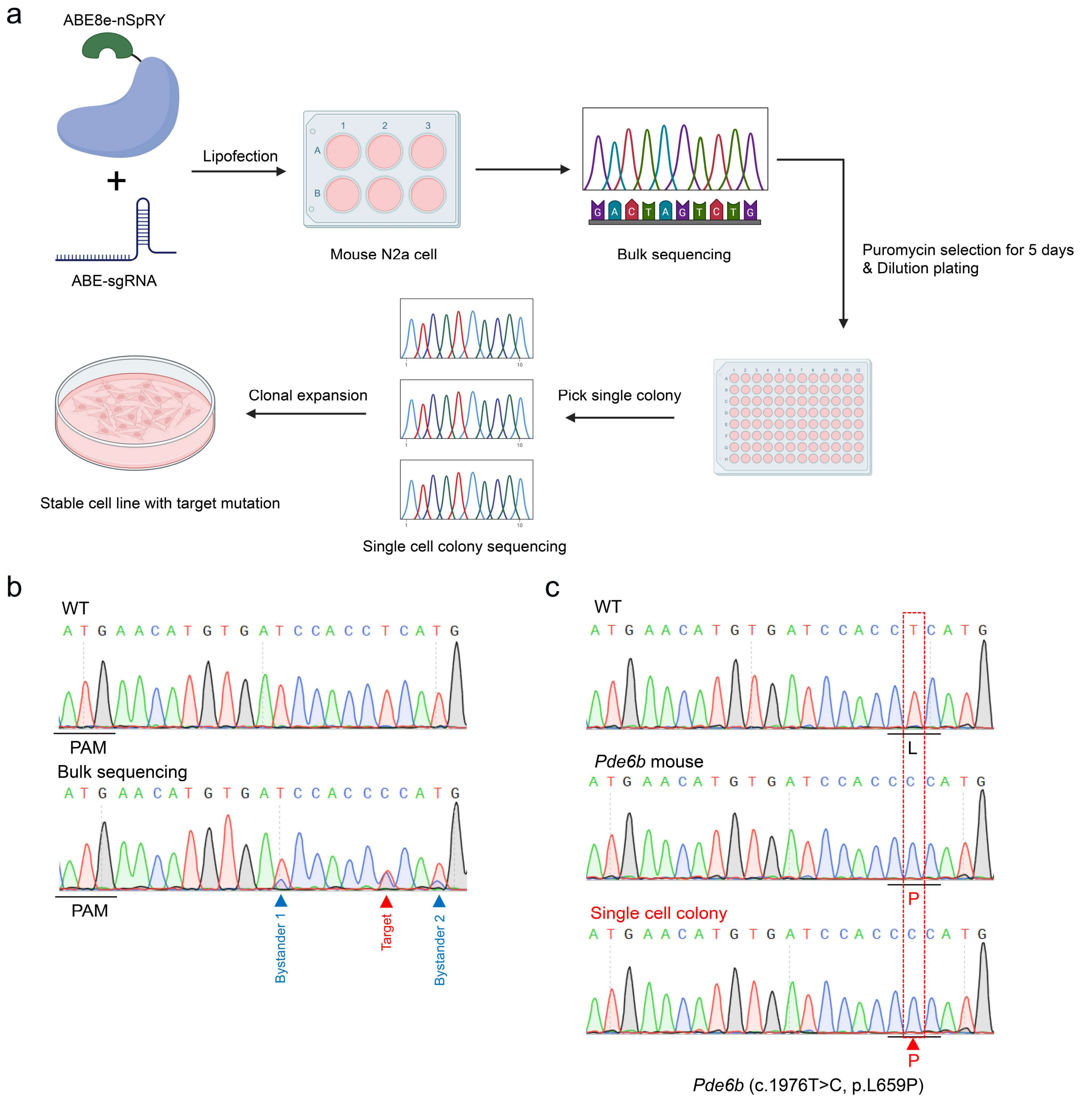

Fig. S1 Generation of a N2a cell line carrying the *Pde6b* (c.1976T>C, p.L659P) mutation. **a** Experimental pipeline of constructing the N2a cell line stably carrying the *Pde6b*-L659P mutation. **b** Sanger sequencing chromatograms of edited bulk N2a cells at the *Pde6b* site. Target editing, red; Bystander editing, blue. **C** Sanger sequencing chromatograms of edited single cell colony with precise homozygous T-to-C mutation at the *Pde6b*-L659P site.

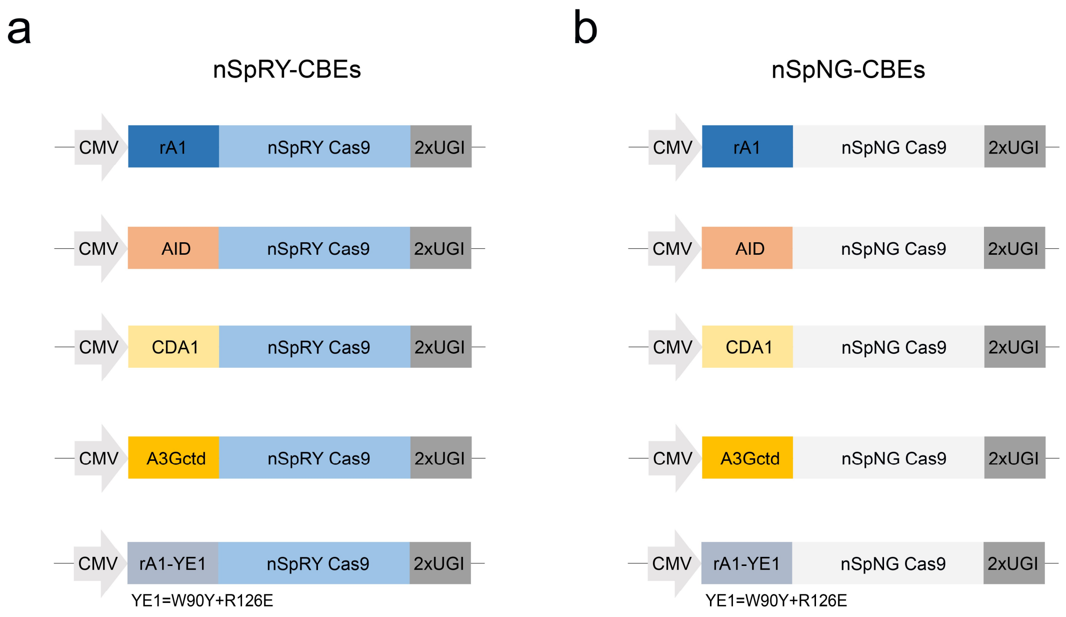

Fig. S2 Schematic representation of constructed nSpRY-CBEs and nSpNG-CBEs used in this study. From top to bottom, the order is rA1-CBEs, AID-CBEs, CDA1-CBE, eA3G-CBEs, and YE1-CBEs. YE1 is derived from rA1 with two point mutations (W90Y + R126E) introduced.

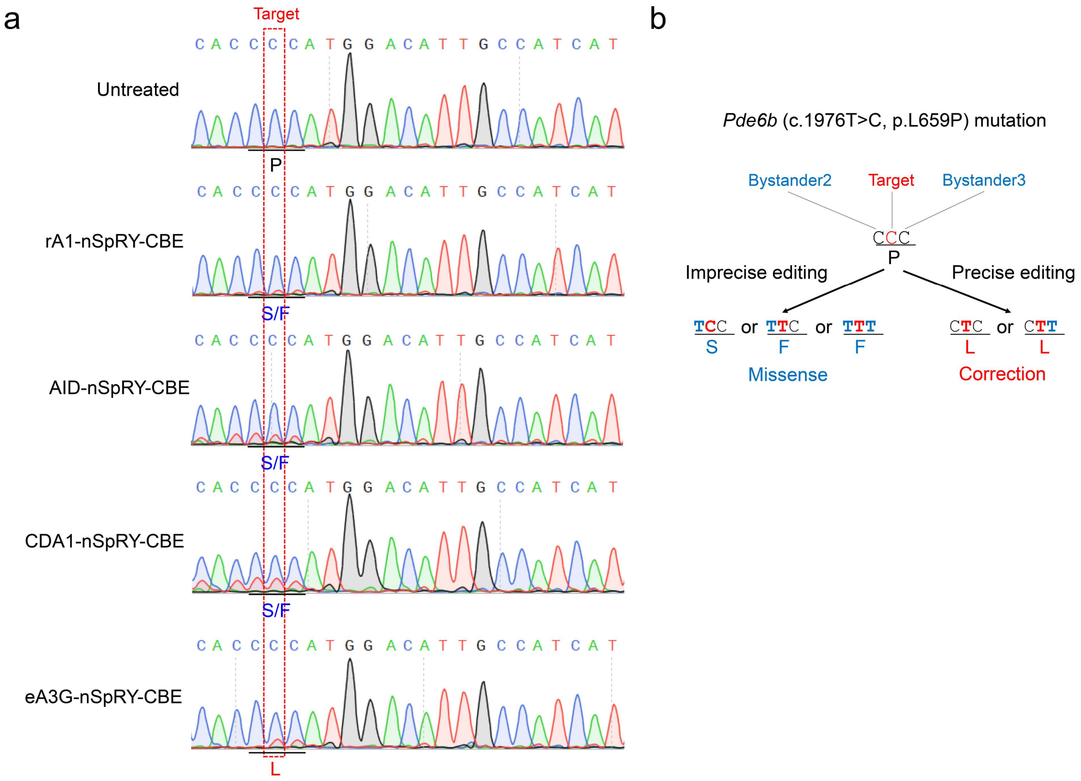

Fig. S3 Editing outcomes and analysis of the CBE systems repairing the *Pde6b*-L659P mutation. **a** Representative sanger sequencing chromatograms of the CBE-edited *Pde6b* site in the N2a cell model. **b** Analyze the impact of bystander mutations on amino acid changes in the *Pde6b*-L659P site. The mutation of bystander2 causes an unintended missense mutation, while the bystander3 mutation does not affect the correct amino acid repair due to codon degeneracy.

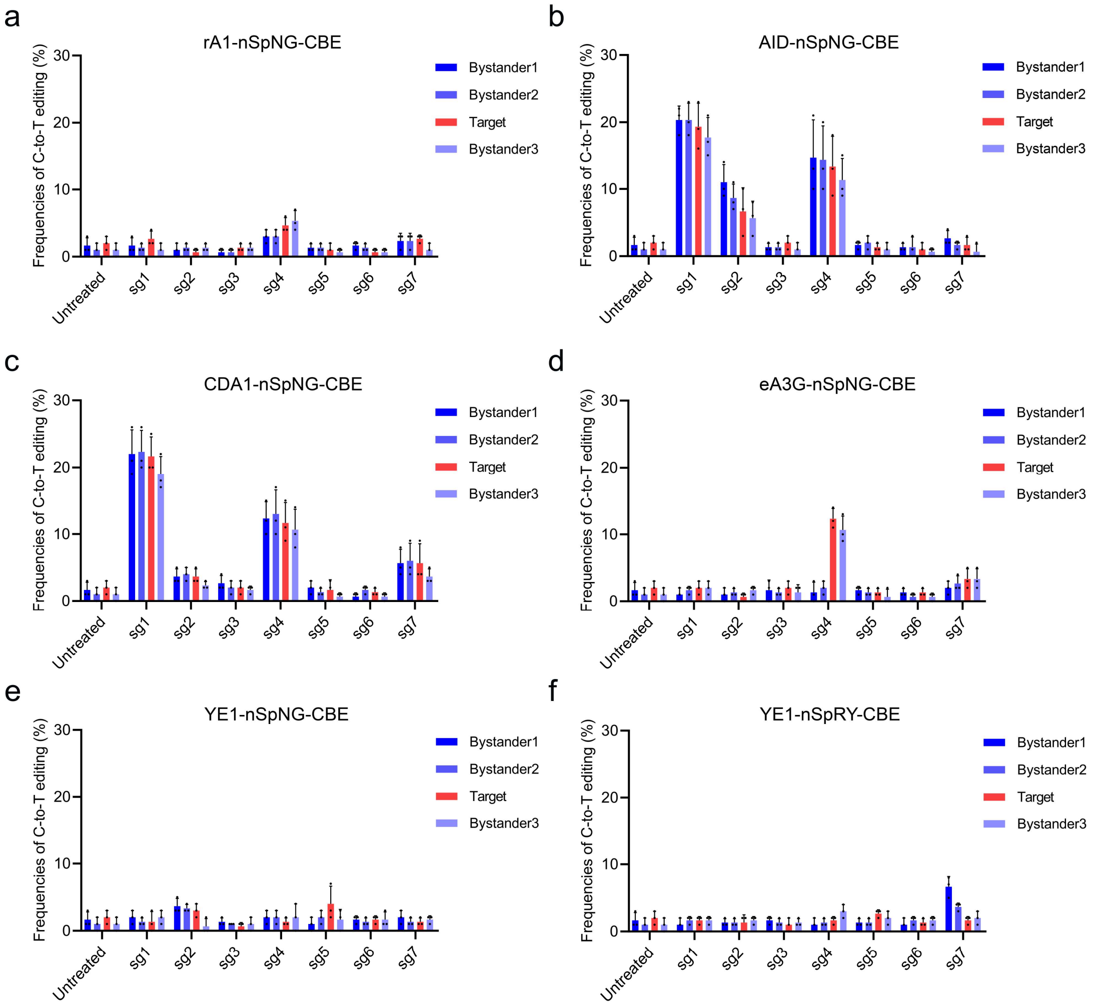

Fig. S4 Editing efficiencies of seven tested sgRNAs with six CBEs in the N2a cell model. (n=3 biologically independent experiments).

a

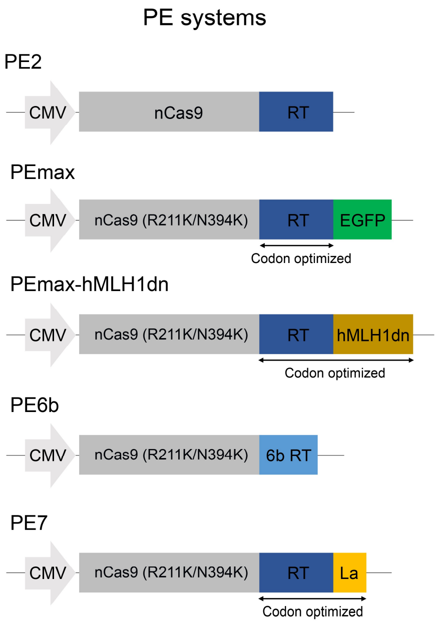

Fig. S5 Schematic representation of five PE vectors used in this study. The conventional PE2 utilized nCas9 (H840A) fused with MMLV reverse transcriptase (RT). PEmax has undergone numerous optimizations compared to PE2, primarily including the introduction of two additional mutations in nCas9 (R211K/N394K) and further codon optimization. PEmax-hMLH1dn incorporated a human dominant negative MMR protein (hMLH1dn) to inhibit cellular MMR based on the PEmax. PE6b uses a designed and optimized 6b RT. PE7, on the other hand, adds a La protein, the small RNA-binding exonuclease protection factor La, to the PEmax framework.

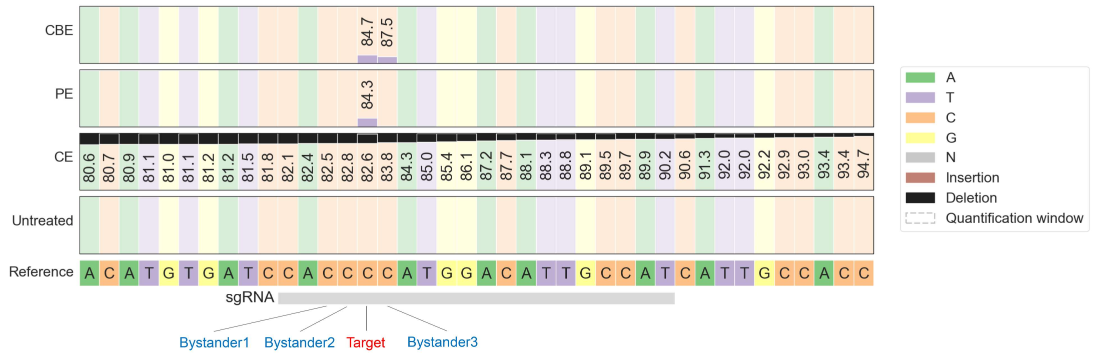

Fig. S6 Heat maps showing all base changes efficiency of CBE, PE and CE systems for *Pde6b*-L659P mutation repair in the N2a cell model. Bystander1-3 represent the C bases surrounding the target C that are easily edited simultaneously, leading to bystander mutations.

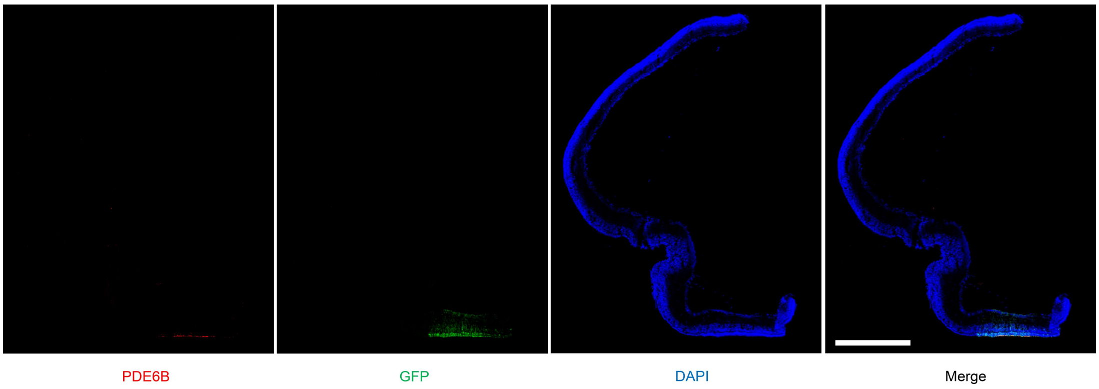

Fig. S7 Representative immunofluorescence images of whole retinal section in PE-treated *Pde6b*-L659P mice at P30. Nuclei are counterstained with DAPI (blue), and GFP signal (green) marks successful electroporation. The PDE6B signal (red) completely overlaps with the GFP signal, indicating that the PE treatment effectively and specifically restored PDE6B expression. Scale bar: 500 μm.

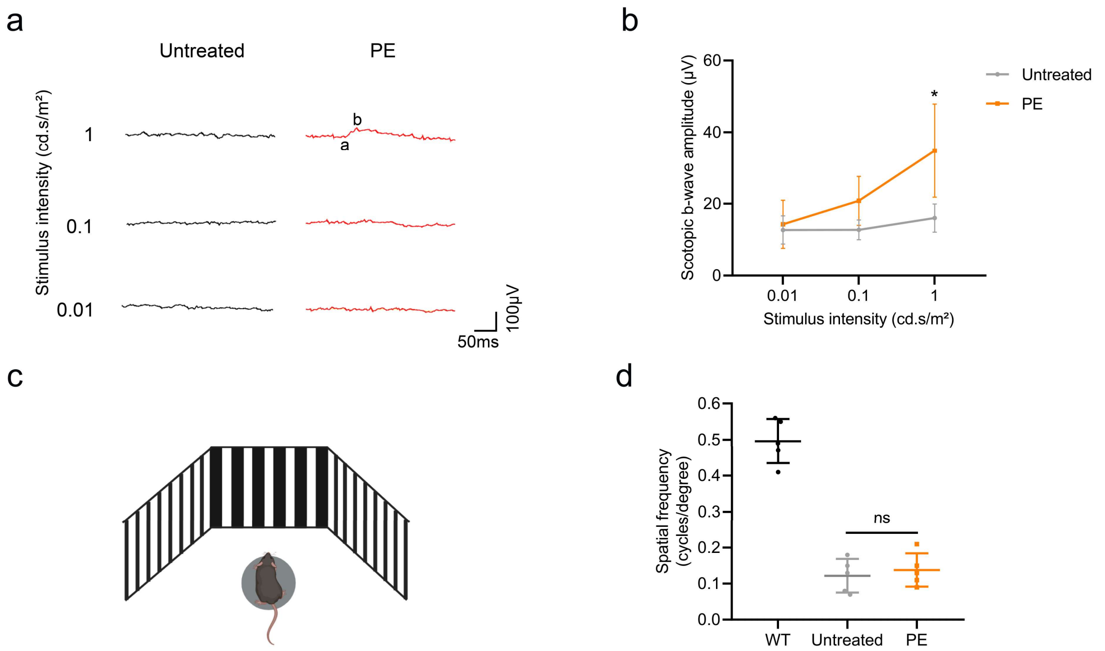

Fig. S8 Evaluation of retinal function in *Pde6b*-L659P mice after PE treatment. **a** Representative scotopic ERG response of untreated and PE-treated *Pde6b*-L659P mice at P30. **b** Quantification of scotopic ERG b-wave amplitudes from each group at P30 (n=5 mice). *$p <$ 0.05. **c** Schematic of optokinetic tracking response (OKR) test in mice. (Created with BioRender.com). **d** Quantification of visual acuity in WT mice, untreated and PE treated *Pde6b*-L659P mice at P30 by OKR testing (n=5 mice).

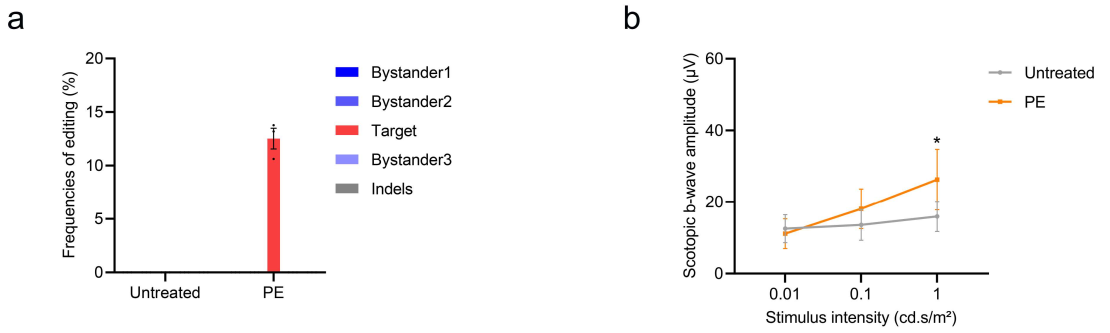

Fig. S9 Evaluation of the long-term PE rescue effect in *Pde6b*-L659P mice at P60. **a** Editing efficiency of untreated and PE-treated mouse retinas at the *Pde6b-L659P* site (n=3 mice). **b** Quantification of scotopic ERG b-wave amplitudes from untreated and PE-treated *Pde6b*-L659P mice at P60 (n=5 mice). *$p < 0.05$.

Raw Images of Figure 6d

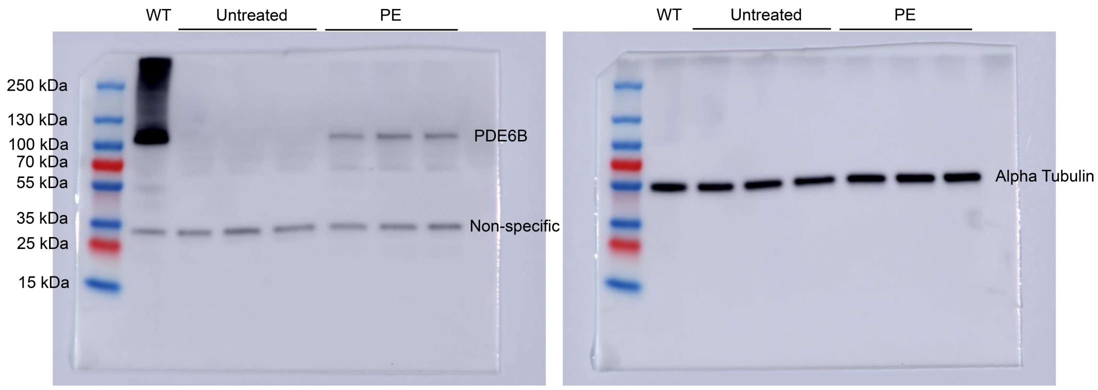

Supplement: Supplementary 1 — Figs. S1 to S9 Tables S1 to S3 [file research.0770.f1.zip › Supplementary information.pdf]
